# WTAP Mediated N6-methyladenosine RNA Modification of ELF3 Drives Cellular Senescence by Upregulating IRF8

**DOI:** 10.7150/ijbs.90459

**Published:** 2024-02-25

**Authors:** Lei Zhou, Yun Zhong, Fan Wang, Yi Guo, Rui Mao, Hongfu Xie, Yiya Zhang, Ji Li

**Affiliations:** 1 Department of Dermatology, Xiangya Hospital, Central South University, Changsha, P.R. China.; 2 Hunan key laboratory of aging biology, Xiangya Hospital, Central South University, Changsha, P.R. China.; 3 Department of Dermatology, the Third Affiliated Hospital, Sun Yat-sen University, Guangzhou, P.R. China.; 4 National Clinical Research Center for Geriatric Disorders, Xiangya Hospital, Central South University, Changsha, Hunan, P.R. China, 410008.

**Keywords:** WTAP, m6A, senescence, ELF3, IRF8

## Abstract

N6-methyladenosine (m6A), the most prevalent posttranscriptional RNA modification, involved in various diseases and cellular processes. However, the underlying mechanisms of m6A regulation in skin aging are still not fully understood. In this study, proteomics analysis revealed a significant correlation between Wilms' tumor 1-associating protein (WTAP) expression and cellular senescence. Next, upregulated WTAP was detected in aging skin tissues and senescent human dermal fibroblasts (HDFs). Functionally, overexpressed WTAP induced senescence and knockdown of WTAP rescued senescence of HDFs. Mechanistically, WTAP directly targeted ELF3 and promoted its expression in an m6A-dependent manner. Exogenous-ELF3 overexpression evidently reversed shWTAP-suppressed fibroblast senescence. Furthermore, ELF3 induced IRF8-mediated senescence-associated secretory phenotype (SASP) by binding to the (-817 to -804) site of the IRF8 promoter directly. *In vivo*, overexpression of WTAP evidently increased senescence cells in skin and induced skin aging. In summary, these findings revealed the critical role of WTAP-mediated m6A modification in skin aging and identified ELF3 as an important target of m6A modification in HDFs senescence, providing a new idea for delaying the aging process.

## Introduction

Skin aging is the most direct manifestation of organism aging, and dryness, sagging, wrinkles, and subcutaneous fat thinning visually reflect an organism's age and potential health status 1. The imbalance of skin tissue homeostasis after aging can affect important pathological and physiological processes such as wound healing, infection, and even tumor occurrence 2. However, the mechanisms of skin aging remain unclear.

Cellular senescence plays an important role in the aging of various organs and bodies 3. Senescent cells have been found to accumulate in the skin with age, leading to skin aging 4. Cellular senescence is referred to as an irreversible state in which cells arrest proliferation. There are various stresses that can cause cellular senescence, including DNA damage, telomere attrition, epigenetic alteration, chronic inflammation, UV radiation, and oxidative stress. In addition to a stable cell cycle arrest, senescent cells also present a set of characteristic phenotypic alterations, including abnormal morphology, increased DNA damage, chromatin remodeling, and aberrant SASP 5. Emerging evidence suggests that cellular senescence regulates various pathophysiological processes, including embryonic development, tissue repair, regeneration, organismal aging, and cancer progression 6-8.

Current studies suggest that cellular senescence is regulated not only genetically but also epigenetically. There is considerable evidence that aging-related diseases are typically characterized by universally epigenetic changes, such as nucleosome positioning, genomic imprinting, DNA modifications, and RNA methylation 9. In recent years, the important role of RNA modification in biological process has attracted much attention. N6-methyladenosine (m6A) is the most extensive post-transcriptional modification in eukaryotic mRNA, participating in various biological events including mRNA transcription, splicing, translocation, positioning and stability 10. The biological functions of RNA m6A are dynamic and reversible, which are accomplished by m6A methyltransferase, demethylases and RNA binding proteins jointly. METTL3, METTL14 and WTAP, as the main components of methyltransferase complex, participated in the catalytic formation of m6A. In addition, several novel proteins (e.g., ZC3H13, RBM15/15B and ZCCHC4) have been identified to play an important role in promoting methyltransferase complex formation 11. The erasers, ALKBH5 and FTO, can effectively reverse this m6A modification process 12-14. Transcripts modified with m6A can be decoded by a family of YTH reading proteins, including YTHDF1-3 and YTHDC1-2 15, 16, and other m6A reader protein, such as FMR1 (fragile X mental retardation 1) and IGF2BP family protein, to perform various biological functions 10, 16. However, the relationship between the m6A modification and aging is poorly studied.

WTAP (Wilm's tumor 1-associating protein) is a conserved nuclear protein and one of the key components of the m6A methyltransferase complex *in vivo*. WTAP plays a critical role in tumorigenesis and antiviral immunity in mammals 17-20. Although WTAP alone did not exhibit methyltransferase activity during *in vitro* assays, the silencing of WTAP markedly diminished the peak of N6-methyladenosine (mA), a reduction that was notably more substantial than that observed with the knockdown of either METTL3 or METTL14 21, 22. Cyclin kinase mRNA has been reported to be regulated by WTAP by enhancing its stability, thereby affecting cell proliferation 23. Recently, researchers found that upregulated WTAP contributed to intervertebral disc degeneration through regulating NORAD m6A modification-mediated senescence of nucleus pulposus cells (NPCs) 24. However, the role of WTAP in skin aging remains unknown.

In the current study, we found the up-regulation of WTAP in aging skin tissues and senescent HDFs, overexpressed WTAP induced HDFs and skin aging. Further functional and mechanistic investigations identified that WTAP increased ELF3 expression in a m6A-dependent manner, subsequently promoted the transcription factor IRF8-indced SASP to accelerate cellular senescence.

## Methods and Materials

### Data acquisition

The proteomics data of human diploid fibroblast from 82 healthy individuals were obtained from MassIVE database (MSV000088401) 25. MeRIP-seq data of WTAP (GSE46705) was downloaded from GEO dataset 26.

### Bioinformatic Analysis

For m6A-related gene expression profile analysis, sixteen widely accepted m6A modification regulators were searched from public research. The heatmap described the expression of m6A RNA methylation regulators in fibroblasts and skin tissues using "pheatmap" packages. The correlation between genes expression and age were descripted by Pearson analysis using “ggpubr”, “ggplot2”, “ggExtra” and "corrplot" packages 27. The genes expression from individuals with high/low p16 were analyzed using "vioplot" packages 28.

Our study utilized single-cell RNA sequencing datasets from public databases. Key dataset details are in Supplementary Table 1 (Appendix). We used Seurat for analysis, starting with quality control, selecting cells based on feature counts and mitochondrial RNA 29. Post integration with Canonical Correlation Analysis, we normalized data, reduced dimensions using UMAP, and clustered. Cell types were identified using specific markers: Macrophages and endothelial cells (IFI27, GNG11), spinous cells (KRT10, KRT1), melanocytes (PMEL, DCT, MLANA), Schwann cells (MPZ, S100B), lymphatic endothelial cells (CLDN5, CCL21), myeloid and dendritic cells (HLA-DRA, HLA-DRB1, IL1B, HLA-DPB1), fibroblasts (APOD, DCN), basal cells (KRT14, KRT5), secretory epithelial cells (AQP5, CA6), pericytes (ACTA2, TPM2), and T, NK, and B cells (CXCR4, LTB, CD3D).

### Skin samples and Immunohistochemistry analysis

All normal human skin sections were obtained from the patients undergoing skin surgery for non-malignant lesions (with the final pathological diagnosis being melanocytic nevus) at the Department of Dermatology of Xiangya Hospital, Central South University. The ethical approval for using human samples in this study was granted by the ethical committee at Xiangya Hospital, Central South University. All participants provided written informed consent. Additionally, our experiments adhered to the ethical standards outlined in the WMA Declaration of Helsinki and the Department of Health and Human Services Belmont Report. The participants were divided into three different groups: young, from 18 to 29 years (n=8; mean age 23.8), middle, from 30 to 69 years (n=12; mean age 42.0), and old, from 70 to 82 years (n=6; mean age 76.5). The information on skin samples from individuals of different ages is listed in Supplementary Table 2 (Appendix). For immunohistochemistry assay, skin sections were deparaffinized, re-hydrated, and recovered with 0.01 M citrate buffer. The anti-WTAP (Abcam, catalog ab195380) were used at 1:1000 overnight at 4°C. The SignalStain® Boost IHC Detection Reagent kit (Cell Signal Technology, USA) was used in this study.

### Cell culture and treatments

Primary HDFs were extracted from the foreskin of healthy 5- to 24-year-old donors and were cultured as described previously 30. Briefly, fresh skin samples were prepared by washing in PBS, removing subcutaneous tissues, and incubating overnight in 5 mg/mL dispase for dermis collection. The dermis was then digested with collagenase IV to obtain a single cell suspension. These dermal cells were cultured and passaged in DMEM medium supplemented with 10% FBS, 1% non-essential amino acids, and 100 U/mL Penicillin-Streptomycin at 37 °C in 5% CO2. Primary HDFs were obtained with written consent from voluntary, informed donors, following a protocol approved by the Clinical Research Ethics Committee at the Xiangya Hospital of Central South University in Changsha, China.

HDFs were continuously passaged, according to the population doubling (PD) differences of cell culture, we defined HDFs < 10 PD as young-passages (Y) cells, defined HDFs with 15-25 PD as middle-passages (M) cells, and defined HDFs > 40 PD as old-passages (O) cells. For the induction of premature senescence, early passage cells at approximately 75-80% confluence was exposed to hydrogen peroxide (200μM for 2 h), UVA (10 J/cm^2^ ×3d), or HRAS oncogene, respectively 31, 32.

Lentiviral shuttle plasmid containing WTAP shRNA or cDNA was co-transfected with other packaging plasmids (VSVG and delta8.9) into HEK293 cells. After 48 hours, the supernatant containing virus was centrifuged, collected and continued to infect HDFs for 2 days. Puromycin was used to continuously screen infected-positive cells. To generate the lentiviral shuttle plasmid, the synthesized short hairpin RNAs (shRNAs) against WTAP (shWTAP#2 and #3) were cloned into pLKO.1 vector, and the WTAP cDNA (LV-WTAP) were cloned into pLVX vector (Primers are listed in Supplementary Table 3 (Appendix)).

### Total RNA extraction and quantitative real-time PCR (qPCR)

Total RNA was extracted from cells using the Trizol^TM^ reagent (Invitrogen, USA). Complementary DNA was synthesized from 1 μg total RNA using the Maxima H Minus First Strand cDNA Synthesis Kit with dsDNase (Thermo Fisher Scientific, USA) according to the manufacturer's instructions. iTaqTM Universal SYBR® Green Supermix (Bio-Rad, USA) was performed for qPCR assay by using CFX Connect Real-Time PCR System (Bio-Rad, USA). The sequences of qPCR primers were shown in Supplementary Table 3 (Appendix).

### Western blotting

The collected cells were lysed in cell lysis buffer (Thermo Fisher Scientific, USA) containing protease inhibitors cocktail (Thermo Fisher Scientific, USA) after being washed with cold PBS buffer. The extracted proteins were quantified via BCA assay (Thermo Fisher Scientific, USA), and heated with SDS loading buffer at 95 °C for 10 min. The pretreated protein samples were separated on SDS- PAGE gel electrophoresis and transferred to PVDF membranes. Then, the membranes were blocked with 5% non-fat milk for 1 h at room temperature and incubated with primary and secondary antibodies respectively. The membranes were detected on ChemiDoc^TM^ XRS+ system (Bio-Rad, USA). Anti-WTAP (1:1,000, Abcam, catalog ab195380), anti-GAPDH (1:5,0000; Proteintech, catalog 60004-1-Ig), anti-p53 (1:1,000; Cell Signal Technology, catalog 2524), anti-p21 (1:1,000; Cell Signal Technology, catalog 2947), anti-p16 (1:1,000; Cell Signal Technology, catalog 18769), anti-p-ATM (1:1,000; Cell Signal Technology, catalog 13050), anti-ATM (1:1,000; Cell Signal Technology, catalog 2873), anti-Bcl-2 (1:1,000; Cell Signal Technology, catalog 15071), anti-Bax (1:1,000; Cell Signal Technology, catalog 41162), anti-ELF3 (1:1,000, Santa cruz, catalog sc376055), anti-IRF8 (1:1,000, Abcam, catalog ab207418).

### RNA m6A quantification analysis and m6A dot blot assay

Total RNA was isolated from the individual dermal fibroblasts with TRIzol^TM^ (ThermoFisher, USA), and then NanoDrop spectrophotometer was used for detecting RNA concentration and quality. The m6A content was quantified by EpiQuik m6A RNA Methylation Quantification Kit (Epigentek, USA) according to the manufacturer's instructions.

For the RNA m6A dot blot assay, total RNA was extracted with TRIzol^TM^ (ThermoFisher, USA) and dripped onto a nylon membrane. After being crosslinked by ultraviolet (UV), the membrane was blocked with blocking buffer and then incubated with anti-m6A antibody (1:1000, Abcam, catalog ab191606) at 4 °C overnight. The m6A dots was analyzed using an imaging system (Bio-Rad, USA).

### Cell proliferation assays

CCK-8 kit (Beyotime biotech, China) was applied to detect cell proliferation according to the manufacturer's protocol. The cells were incubated in CCK-8 diluted with culture media at 37 °C for 2 h, absorbance was determined at 450 nm for 4 consecutive days, respectively.

### Flow cytometry

For the cell cycle assay, the pre-treated cells were collected by centrifugation after trypsinization. Then, the cells were fixed in cold 70% ethanol and incubated at 4 °C overnight. After washing with PBS buffer, cells were resuspended in PBS containing propidium iodide (50 μg/ml) and incubated for 20 min at room temperature. Then, cell cycle analysis was performed on Becton-Dickinson FACS Canto II flow cytometer (Becton-Dickinson, USA).

### Immunofluorescence

In brief, cells seeded on glass coverslips were fixed with 4% paraformaldehyde at room temperature for 10 min and then permeabilized with 0.3% Triton X-100 in PBS for 15 min. After being blocked with blocking buffer (5% normal donkey serum diluted in PBS buffer) for 30 min at room temperature, the cells were incubated with primary antibodies (diluted with 1% donkey serum in PBS) for overnight at 4 °C and then with the fluorescence-conjugated secondary antibodies (Thermo Fisher Scientific, USA) for 1 h at room temperature. DAPI (Thermo Fisher Scientific, USA) was used for nuclear DNA staining. Anti-ki67 (1:8,000, Cell Signal Technology, catalog 9449), anti-γ-H2A.X (1:1,600, Cell Signal Technology, catalog 2577), EdU (Tsingke, China) were used. Images were taken with a LSM 510 Zeiss laser confocal scanning microscope (Carl Zeiss, Germany).

### Senescence-associated β-galactosidase staining

Briefly, after washing with PBS buffer and fixed in 10% formaldehyde at room temperature for 10 min, the cells seeded in six well plates were stained with freshly β-galactosidase staining solution (Beyotime biotech, China) at 37 °C overnight. Images were taken under microscope, and then the quantification of β-galactosidase staining positive cells were calculated and analyzed using ImageJ software (USA).

### RNA-seq analysis

Total RNAs were extracted from stable knockdown cell lines and the controls using TRIzol^TM^ (ThermoFisher, USA) and then genomic DNA was removed using DNase I (Thermo Fisher Scientific, USA). For RNA-seq analysis, the library preparation and high-throughput sequencing were performed by OE Biotech Co., Ltd (Shanghai, China).

### m6A RNA-IP-qRT-PCR (MeRIP-qPCR)

Total RNA was extracted from dermal fibroblasts using TRIzol^TM^ (Invitrogen, USA). Genomic DNA was removed using DNase I (Thermo Fisher Scientific, USA). DNA-free RNA was incubated with magnetic Dynabeads(Abcam, USA) bound to anti-m6A or normal IgG antibodies to precipitate mRNA containing m6A binding sites. For each immunoprecipitation, a total of 500 μg RNA was prepared, and a portion of total RNA was retained as input (Primers are listed in Supplementary Table 3 (Appendix)).

### Luciferase reporter assays

293T cells were seeded in 48-well plates for 24 h, and then each well was transfected with 250 ng of ELF3 vectors, 50 ng of pGL3 plasmid containing IRF8 promoter and 0.25 ng of the Renilla luciferase reporter with Lipofectamine 2000 (ThermoFisher, USA). After 48 h transfection, the cells were harvested. Firefly and Renilla luciferase activities were measured with the Dual-Luciferase Reporter System (Promega, USA).

### m6A methylation site prediction and transcription factor binding site prediction

mRNA sequences were used to predict the putative m6A recognition sites by websites (http://www.cuilab.cn/sramp/). In order to predict the possible results, three types of random forest models were used: binary, KNN, and spectrum.

The feasible binding sites of ELF3 in the IRF8 promoter was predicted by the JASPAR website (http://jaspar.genereg.net/).

### Chromatin Immunoprecipitation (ChIP) Assay

Chromatin immunoprecipitation assays were performed with a Magana CHIP A/G kit (Merck, Germany). In briefly, cells were chemically crosslinked using 1% formaldehyde for 15 minutes at 25℃. After that, cells were lysed in lysis buffers, the DNA fragment was cleaved by enzyme. Anti- ELF3 antibody, or normal IgG was used for enriching the fragment DNA. After that, the enriched DNA was quantified by qPCR (Primers are listed in Supplementary Table 3 (Appendix)).

### Animal experiments and histological examination

For *in vivo* knockdown of target genes in dermis, 10 μl lentivirus (>5 × 10^8^ cfu/ml) was intradermally injected into 6-week-old BALB/c mice dorsal skin once every other day for 8 weeks, skin aging of mouse model was evaluated by skin wrinkle and hair loss. Skin tissues were collected to be washed with PBS buffer, fixed with formalin for 24 h and embedded in paraffin were stained with hematoxylin and eosin (HE) as previously described.

For Masson staining, paraffin-embedded sections were dewaxed and stained with hematoxylin for 10 min. After washed with PBS, the sections were stained using Masson's staining solution for 10 min. Then, after another washing, the sections were soaked in 2% glacial acetic acid and 1% phosphomolybdic acid solution for 2 min in turn. Next, the sections were stained using aniline blue staining solution for 5 min and washed with weak acid working solution for 1 min. Finally, the sections were dehydrated with 95% ethanol and mounted with neutral gum. All images were taken using a Zeiss microscope and analyzed using ImageJ software (USA).

For SA-β-Gal tissue staining, frozen tissue was fixed with 4% PFA for 15 min, followed by overnight staining at 37 ° C using CellEvent Senescence Green Test Kit (Thermo Fisher Scientific, USA), washed with PBS, and stained with DAPI. All images were taken using Zeiss microscopy and analyzed using ImageJ software (USA).

### Statistical analysis

All statistical analyses were conducted with GraphPad Prism version 8 software (GraphPad Software Company, version 8.0.0, San Diego, CA). All data were presented as mean ± SEM of three independent experiments. The unpaired Student's t-test was used to compare the comparison of two groups and one-way analysis of variance (ANOVA) followed by Dunnett's test was used for multiple comparisons. P values < 0.05 was considered statistically significant.

## Results

### WTAP expression was upregulated in human aging skin tissue and senescent fibroblasts

**Figure 1:**
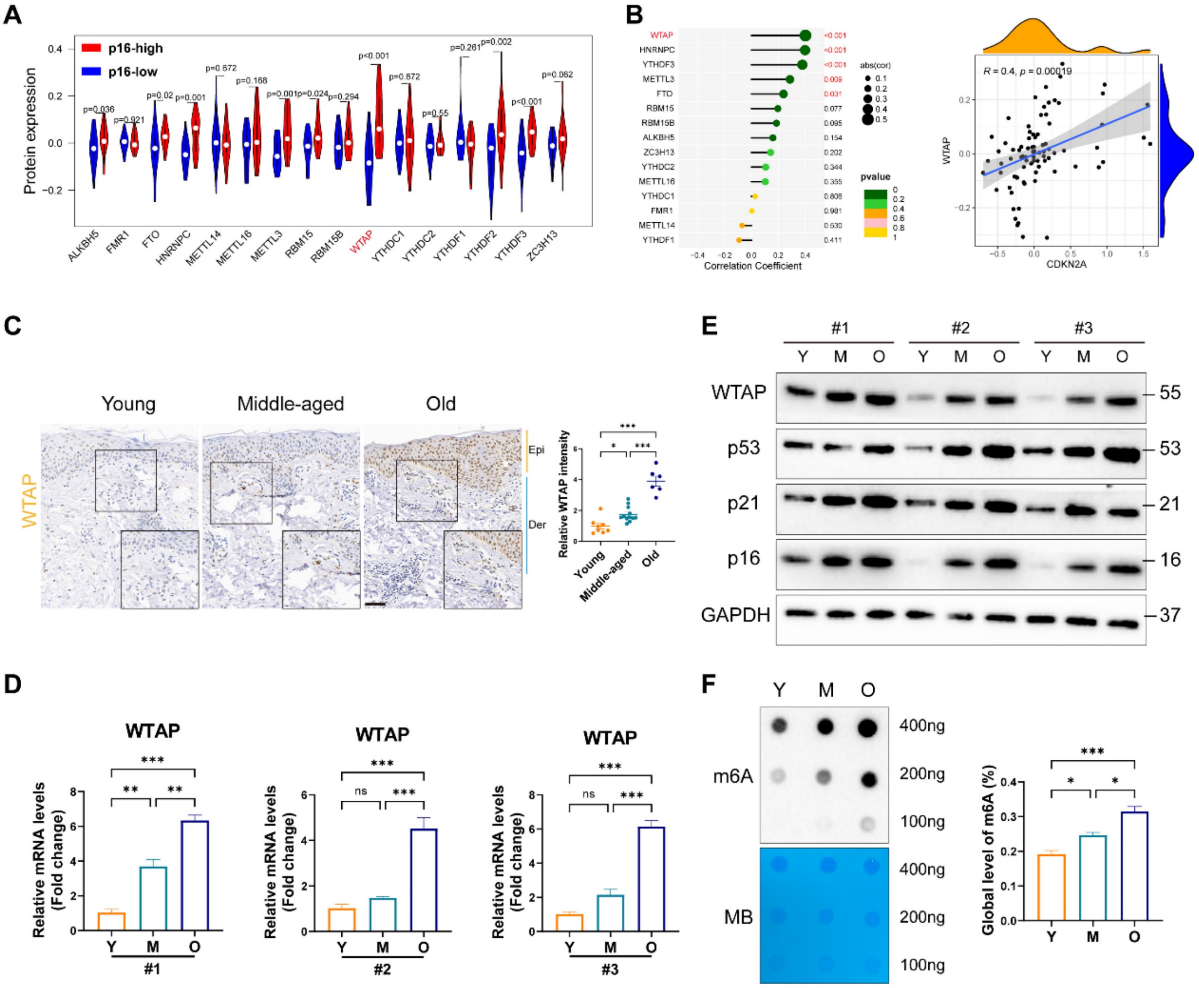
WTAP expression and m6A modification was upregulated in the skin of the elderly and replicative senescent HDFs. (A), The vioplot revealed the protein levels of the m6A regulators in p16-high/low groups. (B), Correlation analysis between m6A regulators protein levels and CDKN2A(p16) protein levels. (C), Immunohistochemistry staining of WTAP in human skin of young (n=8, mean age 23.75), middle-aged (n=12, mean age 42), and old groups (n=6, mean age 76.5). (D), qPCR and (E) western blotting showed the expression of WTAP in young-, middle-, and old-passages HDFs of different individuals. (F), Overall m6A modification in young-, middle-, and old-passages HDFs measured by Dot Blot and m6A colorimetric assay; Methylene blue staining was used as loading control. Data are shown as mean ± SEM. Epi, epidermis; Der, dermis. Scale bars, 50μm. *P < 0.05; **P < 0.01; ***P < 0.001; ns, not significant.

To elucidate m6A modification's role in skin aging, we explored the expression levels of 16 m6A-associated protein in skin fibroblasts using the proteomics data from MassIVE database 25. As p16 (CDKN2A) is a classic biomarker of senescent cells 33, we analyzed the levels of m6A-associated protein between p16-high/-low groups. As shown in Figure 1A, the protein levels of WTAP and YTHDF2 were significantly upregulated in p16-high fibroblasts. Next, Pearson analysis was used to assess the correlation between m6A-related protein and p16 protein levels. We found that WTAP was the most significantly associated with p16 expression (R= 0.4, p= 0.00019) (Figure 1B). Considering the important role of m6A writers, especially WTAP, in diverse biological processes, WTAP was chosen for further analysis. Additionally, single-cell data analysis also revealed that WTAP is variably expressed in various skin cells types, such as keratinocytes, melanocytes, immune cells, and fibroblasts, with a particularly prominent expression in fibroblasts (Figure S1).

Then, we examined WTAP expression in human skin samples of the young, the middle and the elder from Xiangya Hospital of Central South University. Similar to the results from proteomics data, immunohistochemistry staining results showed that WTAP was significantly highly expressed in human skin aging tissues (Figure 1C). Additionally, considering that photoaging is also an important mechanism leading to skin aging, we compared WTAP expression in photoaged skin tissues from exposed and non-exposed areas. Our findings indicated that WTAP expression was elevated in photoaged skin tissues from the exposed areas (Figure S2). Moreover, the mRNA and protein levels of WTAP were much higher in old-passages HDFs compared with that in the young- or middle-passages HDFs (Figures 1D and 1E). Considering the role of WTAP in m6A modification, we detected the total m6A levels in senescent HDFs. At shown in Figure 1F, the m6A level was significantly increased in the HDFs from old-passages group compared to that from middle/young-passages groups. The up-regulation of WTAP expression at the RNA (Figure S3A) and protein (Figure S3B) levels were also observed in UVA or H_2_O_2_-induced senescence HDFs. Meanwhile, the levels of m6A modification also increased in UVA or H_2_O_2_-induced senescence HDFs (Figures S3C). However, in the cellular senescence model induced by HRAS gene, this imagination was not observed (Figures S3C and S3D). Taken together, these findings suggested that WTAP was highly expressed in human aging skin tissues and senescent HDFs which may be associated with aging progression.

### WTAP is essential for cellular senescence

**Figure 2:**
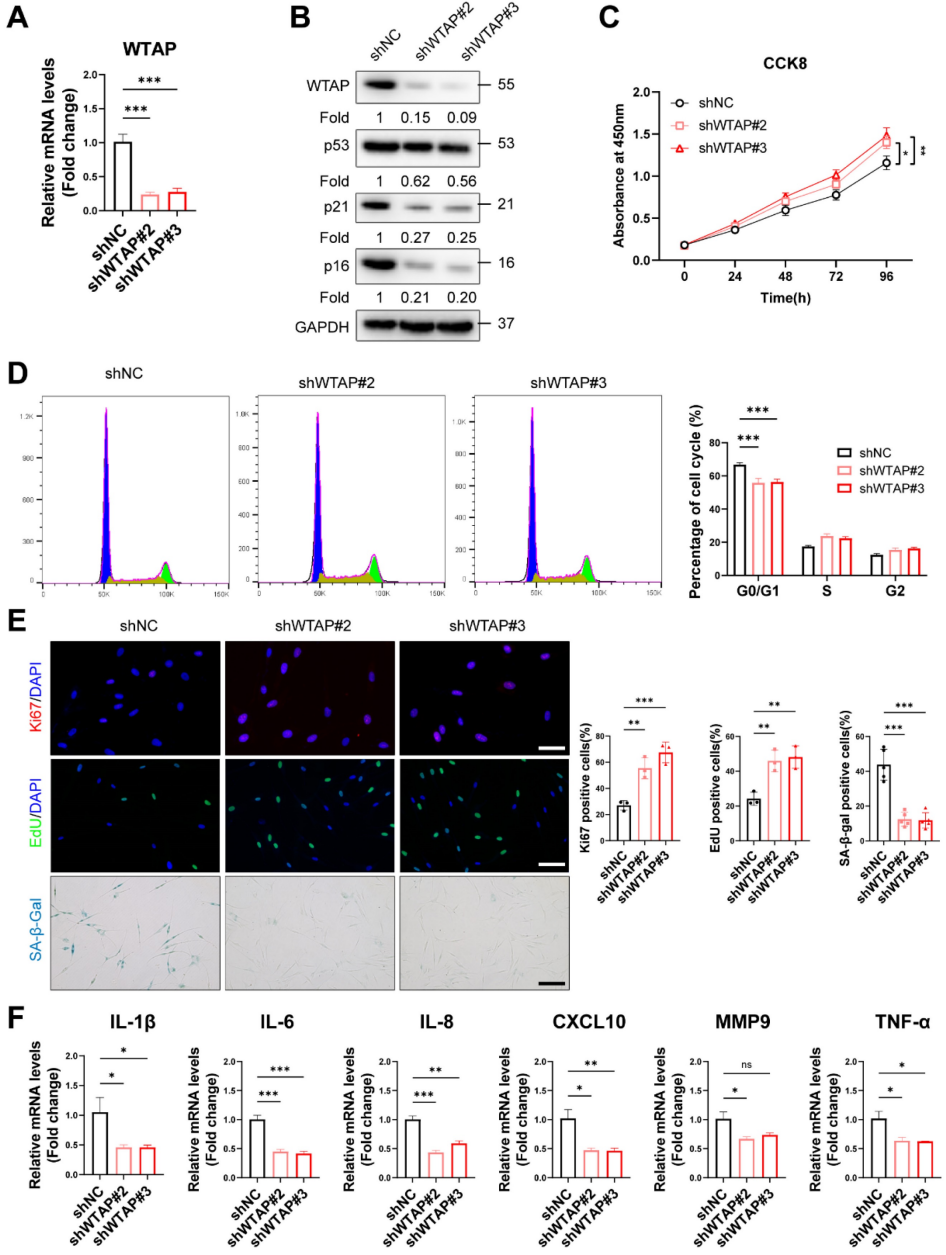
Knockdown of WTAP rescued cellular senescence in senescent HDFs. Senescent HDFs (PD > 45) were transfected with shWTAP or negative control shNC (A), The shWTAP downregulated the mRNA expression of WTAP. (B), The proteins levels of WTAP, p53, p21, and p16 by western blotting. (C), Cell proliferation measured by CCK8. (D), Cell cycle analysis measured by flow cytometry. (E), Immunofluorescence staining of Ki67 and EdU, SA-β-Gal staining in HDFs upon shRNA-mediated knockdown of WTAP. Scale bar, 50 μm. (F), The mRNA expression levels of IL-1β, IL-6, IL-8, CXCL10, MMP3 and TNF-α by RT-qPCR. Data are shown as mean ± SEM. *P < 0.05; **P < 0.01; ***P < 0.001; ns, not significant.

To explore the role of WTAP in HDFs senescence, we silenced WTAP in senescent HDFs (PD > 45). RT-qPCR (Figure 2A) and western blot analysis (Figure 2B) showed that Lentivirus-shWTAP significantly reduced the expression of WTAP in the HDFs. Knockdown of WTAP significantly alleviated senescent phenotype, including p21 and p16 levels (Figure 2B), cell proliferation (Figure 2C), cell cycle arrest (Figure 2D), Ki67 immunoreactivity, EdU incorporation, and SA-β-Gal staining (Figure 2E). Moreover, WTAP knockdown decreased the levels of SASP (IL-1β, IL-6, IL-8, CXCL10, and TNF-α) (Figure 2F).

Subsequently, we tested whether cellular senescence could be accelerated by exogenous WTAP in the young HDFs. RT-qPCR (Figure S4A) and western blot analysis (Figure S4B) showed that lentivirus containing WTAP cDNA (LV-WTAP) significantly increased the expression of WTAP in the HDFs. Overexpressed WTAP significantly aggravated senescent phenotype, including p53, p21, and p16 expression (Figure S4B), cell proliferation (Figure S4C), cell cycle arrest (Figure S4D), Ki67 immunoreactivity, EdU incorporation and SA-β-Gal staining (Figure S4E). Interestingly, overexpressed WTAP also altered the p-ATM level (Figure S4B), which is the marker of DNA damage response (DDR). We next determined whether WTAP-mediated cellular senescence induced the occurrence of DDR. γ-H2AX foci were increased in LV-WTAP infected cells (Figure S4E). To exclude the possible role of apoptosis on cell proliferation, we measured the expression of Bcl-2 and Bax in HDFs. And found that overexpressed WTAP did not alter Bcl-2 and Bax expression in HDFs (Figure S4F). RT-qPCR analysis showed that the mRNA levels of SASP (IL-1β, IL-6, IL-8, CXCL10, MMP3 and TNF-α) were increased in WTAP overexpression group (Figure S4G).

In conclusion, these findings demonstrated that increased WTAP expression is essential for cellular senescence.

### ELF3 was a potential target of WTAP and regulated by WTAP via m6A dependent manner in HDFs

To better explore the potential mechanism of WTAP in cellular senescence, RNA-seq was performed in HDFs with WTAP knockdown. The results showed 1002 downregulated and 247 upregulated DEGs with |log2FC|>1 and adj.p<0.05 in WTAP knockdown group compared with control group (Figure S5A and S5B). GO analysis suggested that DEGs were enriched in extracellular matrix formation and cell adhesion function, and so on (Figure S5C). KEGG pathway analysis revealed that DEGs were enriched in cell adhesion molecule expression, purine metabolism, and complement and coagulation cascades related signal pathway (Figure S5D). In addition, genes in transcription factor families such as Fox, Homeobox, and ETS were also significantly altered after WTAP knockdown (Figure S5E).

**Figure 3:**
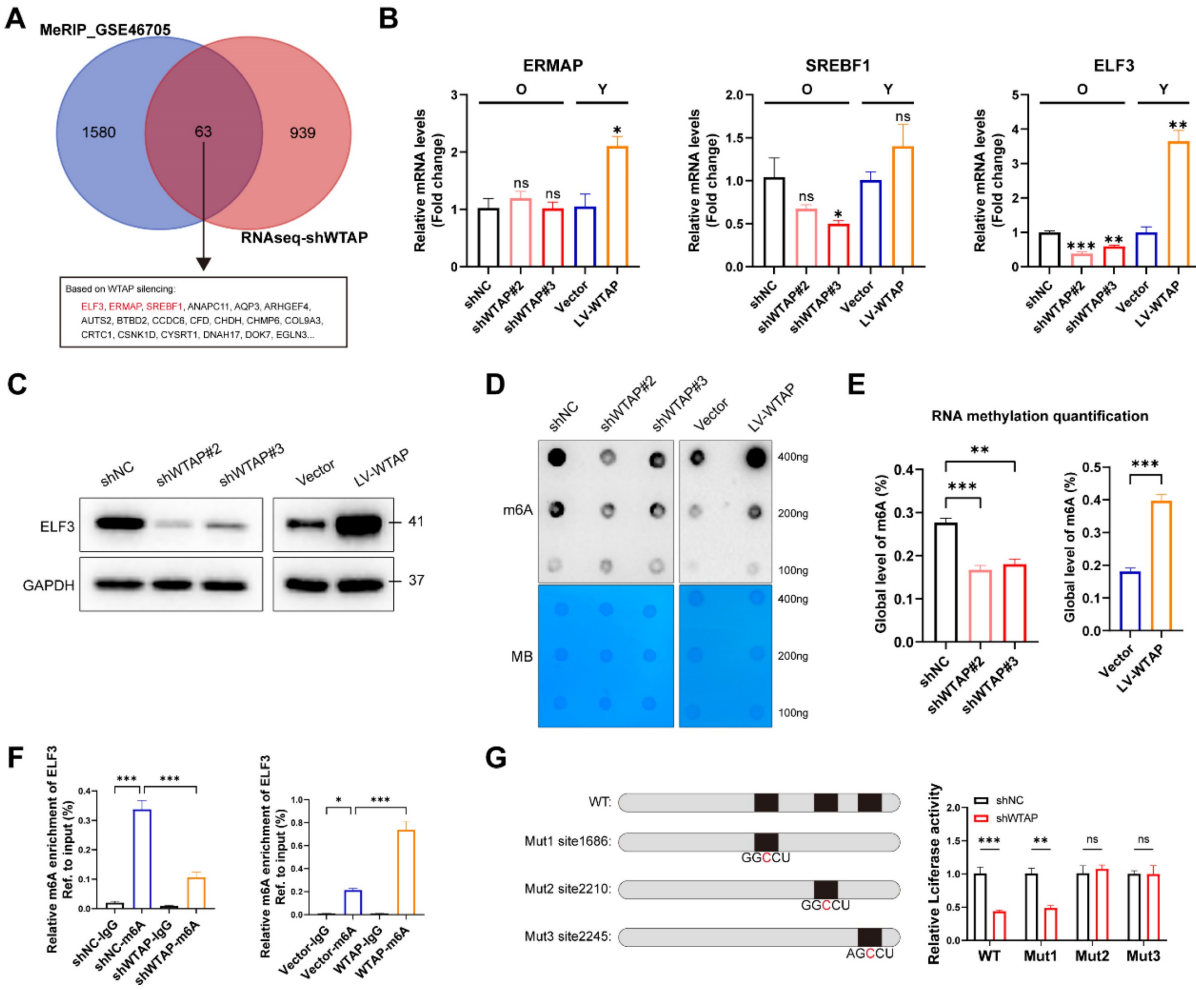
ELF3 was a potential target of WTAP and regulated by WTAP via m6A dependent manner in HDFs. (A), The Venn was derived from the DEGs in m6A-seq in GSE46705 and RNA-seq after WTAP knockdown. 63 genes transcripts were influenced by WTAP according to the overlaps. (B, C), Expression of ERMAP, SREBF1, and ELF3 was examined by RT-qPCR and western blotting in HDFs following the knockdown or overexpression of WTAP. Overall m6A modification in WTAP knockdown or overexpression HDFs measured by Dot Blot (D) and m6A colorimetric assay (E); Methylene blue staining was used as loading control. (F), MeRIP analysis combined with RT-qPCR was used to detect the m6A modification of ELF3 in WTAP-silencing or overexpressing HDFs. The relative m6A enrichment in each group was calculated by m6A-IP/input and IgG-IP/input. (G), Wild-type or m6A site mutant ELF3 were cloned in pGL vector. Luciferase reporter assays presented the target regions of WTAP on the 3′UTR of ELF3. Data are shown as mean ± SEM. *P < 0.05; **P < 0.01; ***P < 0.001; ns, not significant.

In order to confirm the potential target directly regulated by WTAP-mediated m6A modification, we integrated DEGs in RNA-seq with potential targets in MeRIP-seq (GSE46705). In total, we obtained 63 overlapping genes, of which ELF3, ERMAP and SREBF1 were transcription factors (Figure 3A). Figure 3B and 3C showed that knockdown or overexpression of WTAP regulated the mRNA and protein levels of ELF3 in HDFs. ELF3 (E74 like ETS transcription factor 3), a member of the ETS transcription factor family, involved in several physiological and pathological processes, including tumorigenesis, inflammation and immune regulation 34, 35. However, the specific role of ELF3 in cellular senescence remains unclear. Here we found that the expression of ELF3 in senescent HDFs was also significantly higher than that in the young or middle-passages HDFs (Figure S6A). Consistently, RT-qPCR assays also revealed increased expression of ELF3 in different cellular senescence models (Figures S6B and S6C). Based on the above results, ELF3 may be a key downstream target gene mediating the WTAP-induced cellular senescence.

As previous studies revealed that WTAP is involved in the composition of m6A writer complex, which mediates N6 methyladenosine (m6A) methylation of RNAs. Next, we analyzed whether WTAP regulate ELF3 expression depending on m6A modification. It's consistent with our speculation that the total m6A levels of HDFs were remarkably decreased in WTAP-knockdown HDFs and increased in WTAP-overexpression HDFs compared with control HDFs (Figures 3D and 3E). In addition, MeRIP-qPCR results suggested that the m6A levels of ELF3 was remarkably reduced by WTAP knockdown and significantly upregulated by WTAP overexpression (Figure 3F). Three m6A methylation sites in ELF3 mRNA were predicted by SRAMP website (Figure S7). To confirm the effect of m6A modification on ELF3, the predicted adenosine base (A) at the m6A site was mutate to cytosine base (C), and luciferase reporter assays was performed. We found that WTAP knockdown decreased the luciferase activity in WT and Mut1 groups, but do not affect the luciferase activity in Mut2 and Mut3 groups (Figure 3G). Therefore, we suggested that WTAP induced ELF3 expression in an m6A-dependent manner.

### ELF3 was involved in WTAP-mediated cellular senescence in HDFs

**Figure 4:**
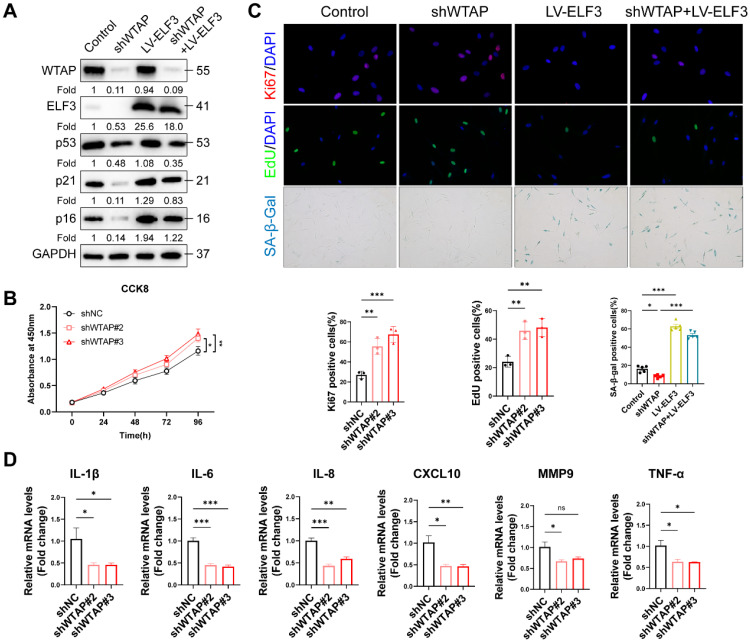
ELF3 was involved in WTAP-mediated cellular senescence in HDFs. (A), Rescue assay was used to assess the reversed effect of ELF3 in WTAP-mediated senescence. The expression of p16 and other senescence-associated proteins was verified at the protein level. CCK8 assay (B), immunofluorescence staining, and SA-β-Gal staining (C) indicated that WTAP knockdown could reverse the cell cycle arrest in ELF3-overexpressed HDFs. (D), The mRNA expression levels of IL-1β, IL-6, IL-8, CXCL10, MMP3 and TNF-α by RT-qPCR. Data are shown as mean ± SEM. *P < 0.05; **P < 0.01; ***P < 0.001.

To reveal the role of ELF3 in WTAP-induced cellular senescence, we overexpressed ELF3 in WTAP knockdown HDFs. We found that exogenous ELF3 was sufficient to rescue the inhibition of silenced WTAP on the expression of senescence-associated markers (p53, p21 and p16) (Figure 4A). The CCK8 and immunofluorescence results showed that overexpressed ELF3 inhibited shWTAP-mediated the increase of cell proliferation, EdU incorporation, and Ki67 immunoreactivity (Figures 4B and 4C). Consistent with the above results, the reduction of SA-β-gal staining and SASP by silenced WTAP was significantly inversed by ELF3 overexpression (Figure 4C and D). Taken together, these results suggested that ELF3 mediated WTAP-induced cellular senescence in HDFs.

### IRF8 was direct transcriptional target of ELF3

**Figure 5:**
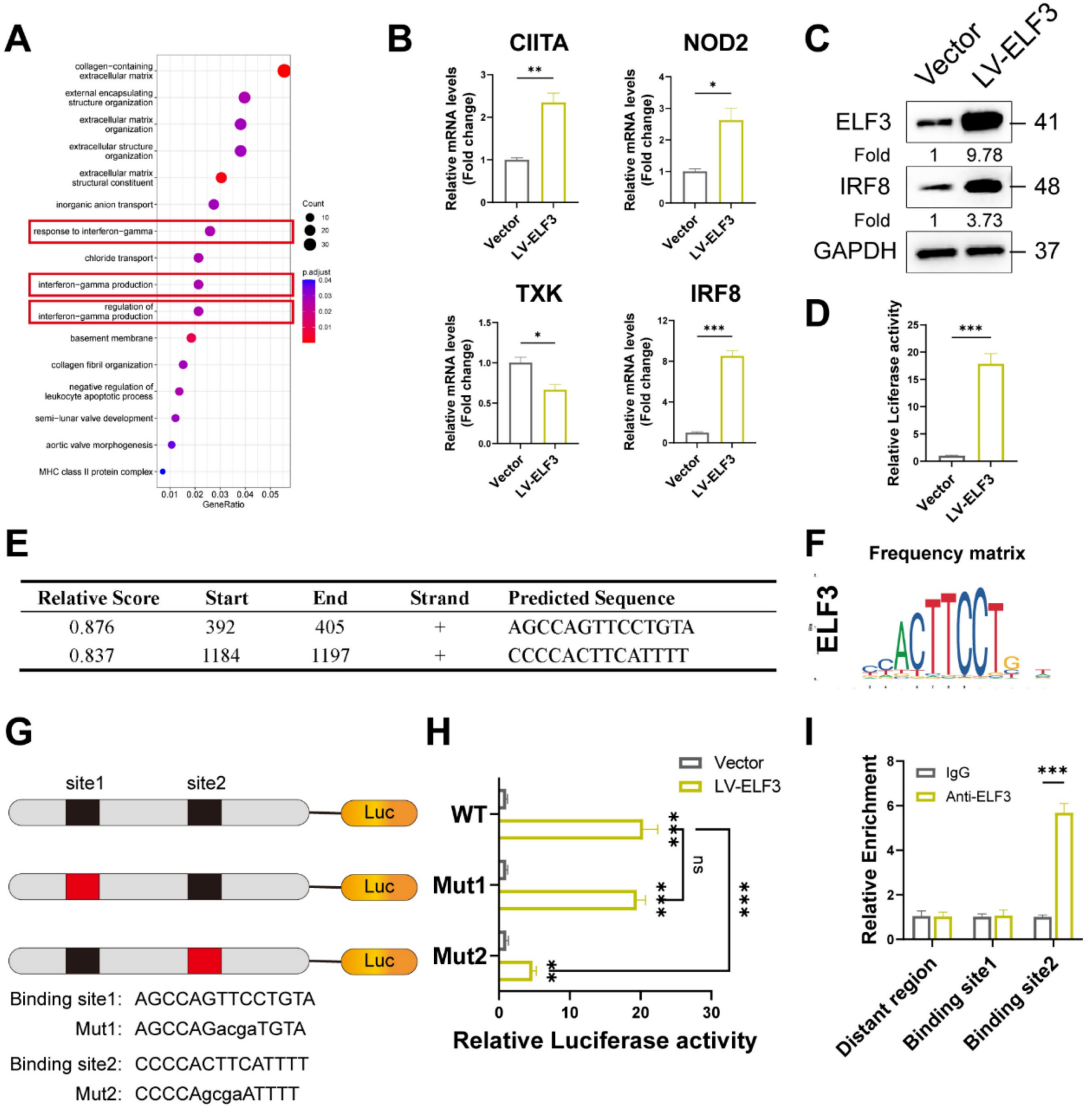
IRF8 was direct transcriptional targets of ELF3. (A), GO pathway revealed the DEGs regulated by ELF3. (B, C), Expression of CIITA, NOD2, TXK, and IRF8 was examined by RT-qPCR and western blotting in HDFs following the overexpression of ELF3, respectively. (D), The IRF8 promoter plasmid was co-transfected with pLKO.1-ELF3, and relative promoter activity was determined using dual-luciferase reporting assay. (E), The 2,000 bp-length of IRF8 promoter was analyzed by JASPAR web tool to predicted possible ELF3 binding sites. (F), The frequency matrix of ELF3 binding sequence was obtained from JASPAR. The wild-type (full-length) or mutant IRF8 promoter plasmids were co-transfected with pLKO.1-ELF3, and relative luciferase activity was detected. The schematic graph is represented (G), relative luciferase activity in each group were presented in the bar graphs(H). (I), ChIP-RT-qPCR results showed that ELF3 was directly bound to the IRF8 promoter in HDFs. Data are shown as mean ± SEM. *P < 0.05; **P < 0.01; ***P < 0.001.

To investigate the potential mechanism of WTAP/ELF3 induces cellular senescence, we analyzed the potential target genes of ELF3 that were differentially expressed in shWTAP RNA-seq data. GO analysis showed that these the potential target genes of ELF3 were enriched in extracellular matrix organization, interferon-gamma production, and so on (Figure 5A). Considering the important role of interferon signaling in cellular senescence 36, 37, we focused on interferon signal associated genes for further study. Among these genes, IRF8 (Interferon regulatory factors 8) was the most significantly upregulated by ELF3 (Figure 5B). The upregulated protein levels of IRF8 were also observed in ELF3 overexpression group (Figure 5C). Moreover, the expression of IRF8 in the senescent HDFs was consistently increased compared to the young or middle-passages HDFs, as verified by western blot analysis and RT-qPCR assays (Figures S4A and S4D). Similarly, relatively high IRF8 expression was detected in UVA, H_2_O_2_ and HRAs-induced senescence HDFs (Figure S4E). After that, the luciferase reporter analysis showed that ELF3 upregulation could enhanced the luciferase activity of the IRF8 promoters (Figure 5D). Two potential ELF3 binding motifs located in IRF8 promoters were predicted by JASPAR web server (Figures 5E and 5F). In order to determine the identified ELF3 binding sites on the IRF8 promoter, we constructed reporter containing different 5 'deletions of the IRF8 promoter. The results revealed that the putative ELF3 binding sites located between -817 and -804 decreased ELF3-mediated activation of the IRF8 promoter (Figures 5G and 5H). Furthermore, the ChIP assay showed that ELF3 was enriched in the region near the IRF8 promoter -817 to -804 in HDFs (Figure 5I). In summary, these results suggested that IRF8 was a direct transcriptional target of ELF3.

### ELF3 induced cellular senescence in HDFs by upregulating IRF8 expression

**Figure 6:**
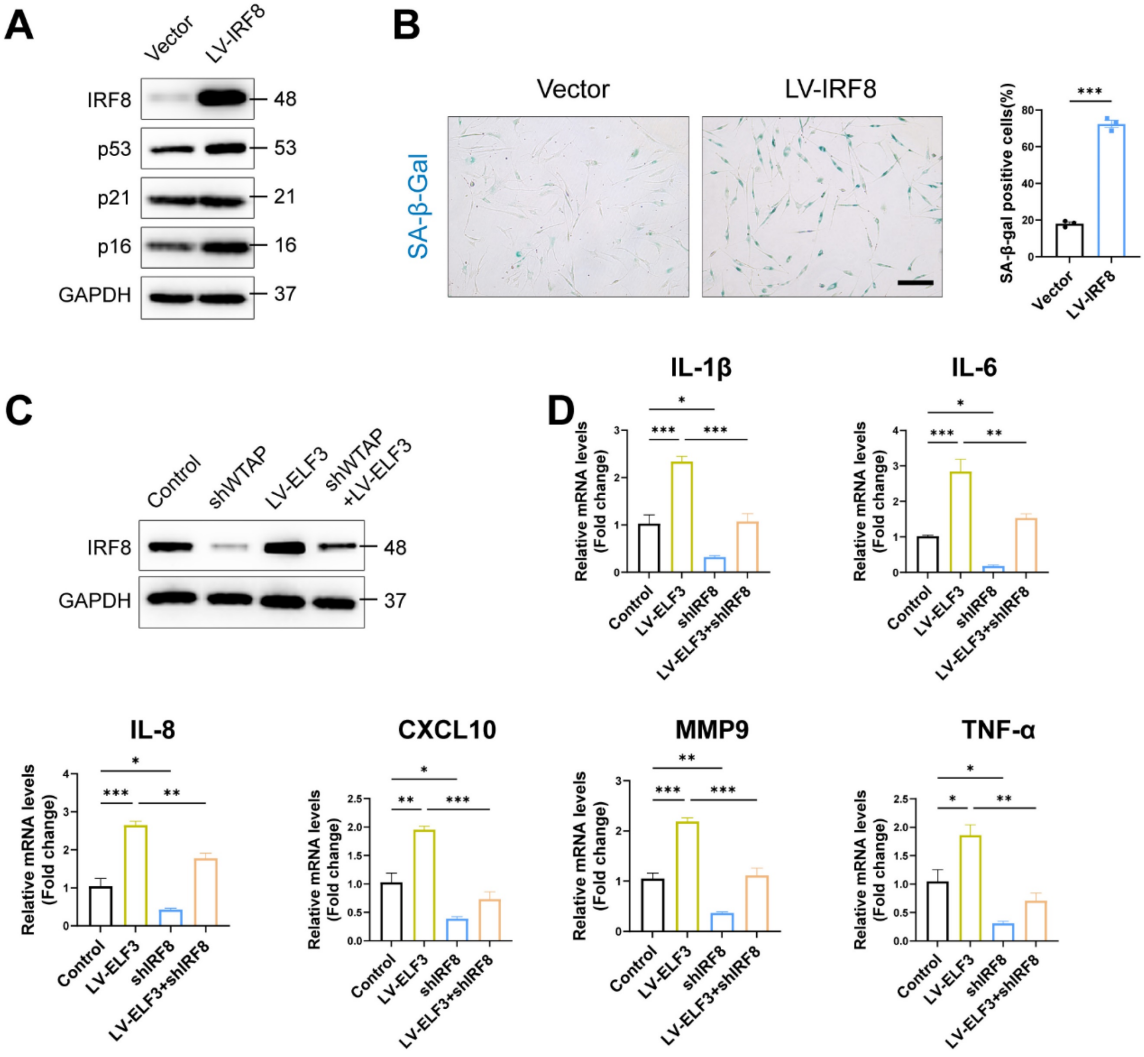
ELF3 induced cellular senescence in HDFs by upregulating IRF8 expression. (A), The LV-IRF8 upregulated the proteins levels of WTAP, p53, p21, and p16. (B), SA-β-Gal staining in HDFs upon lentivirus-mediated overexpression of WTAP. Scale bar, 50 μm. (C), The protein expression level of IRF8. (D), The mRNA expression levels of IL-1β, IL-6, IL-8, CXCL10, MMP3 and TNF-α by RT-qPCR. Data are shown as mean ± SEM. *P < 0.05; **P < 0.01; ***P < 0.001.

IRF8, an important member of the interferon regulatory factor family, involves the regulation of interferon expression, playing an important role in DNA damage-induced cellular senescence 38. Consistent with previous results, exogenous IRF8 significantly induced cell senescence by upregulating the p53 and p16 expression and increasing SA-β-Gal-positive staining in HDFs (Figures 6A and 6B). Next, we explored whether IRF8 mediates WTAP/ELF3-induced senescence in HDFs cells. Western blotting showed that WTAP knockdown downregulated the protein level of IRF8, and overexpression of ELF3 reversed the decrease caused by silencing WTAP (Figure 6C). As interferon signaling pathway related genes mediated senescence-associated secretory phenotype (SASP) in cellular senescence and IRF8 was key inducer of interferon genes 38, we analyzed the role of IRF8 on ELF3-mediated SASP in senescence HDFs. Our results showed that the expression of SASP in HDFs was significantly reduced by silenced IRF8. Meanwhile, the downregulation of IRF8 significantly suppressed the increase of SASP expression caused by ELF3 overexpression (Figure 6D). Therefore, these results implied that ELF3 induced the cellular senescence through IRF8-mediated SASP in HDFs.

### WTAP Overexpression in skins aggravated mouse skin aging

**Figure 7:**
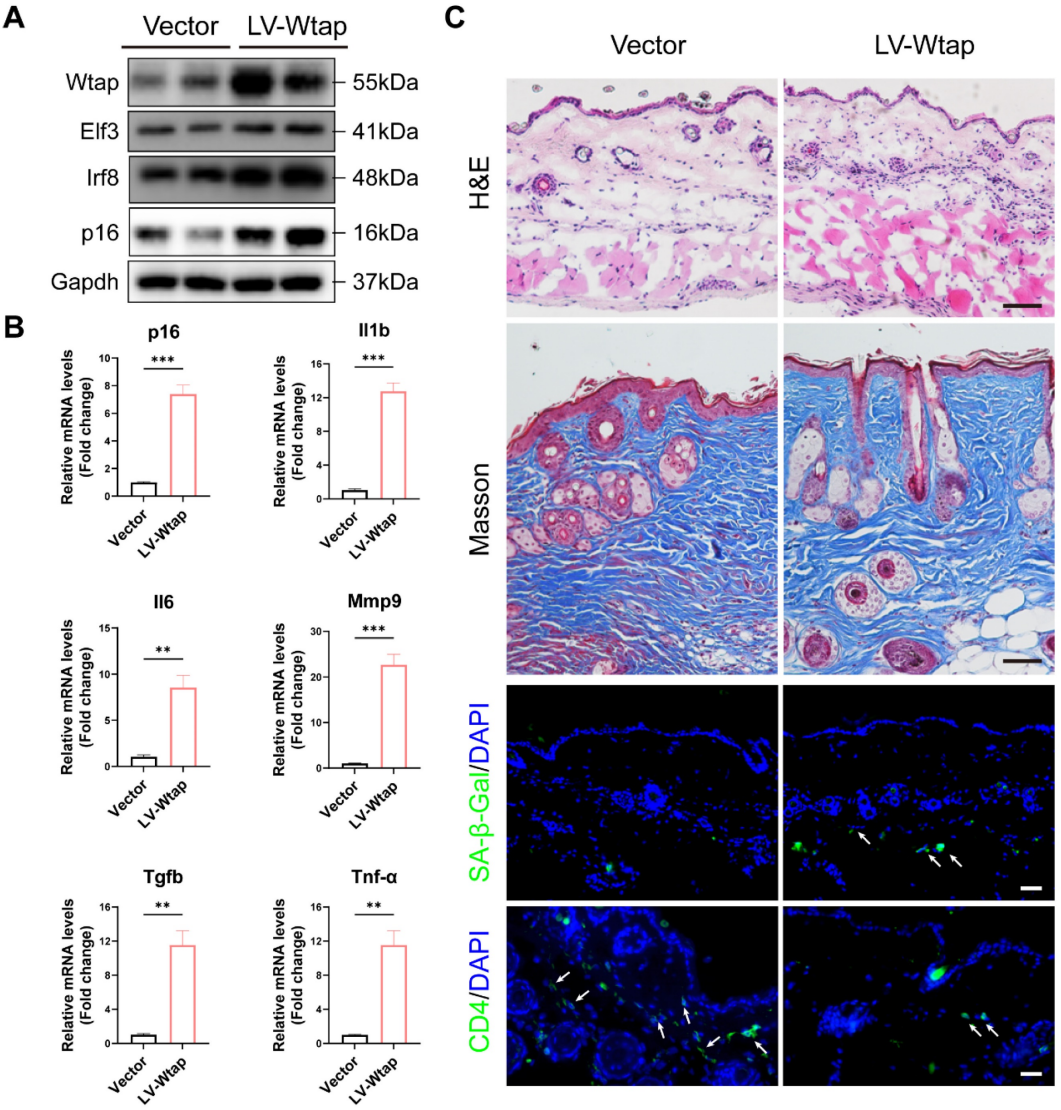
WTAP Overexpression in skins aggravated mouse skin aging. 6-week-old BALB/c mice dorsal skin were injected with 10 µL lentivirus containing Wtap (LV-Wtap, >5 × 108 cfu/ml) or vector once every other day for 8 weeks and sacrificed. (A), The protein expression levels of Wtap, Elf3, Irf8 and p16. (B), The mRNA expression levels of p16, Il1b, Il6, Mmp9, Tgfb and Tnf-α by RT-qPCR. Data are shown as mean ± SEM. (C), H&E staining, Masson staining, SA-β-Gal staining and immunofluorescence staining of CD4 in skin tissues. Scale bar: 50 μm. *P < 0.05; **P < 0.01; ***P < 0.001.

To confirm the role of WTAP in the skin aging, lentivirus LV-Wtap was injected into the mice dorsal skin, and lentivirus containing vector was injected as a control. Our results showed that overexpressed WTAP increased the levels of Elf3, Irf8, p16, and SASP also increased significantly in skin (Figures 7A and 7B). Moreover, as shown in Figure 7C, overexpressed Wtap evidently induced skin aging. Reduced skin thickness and collagen fibers, as well as increased immune cell infiltration within the dermis, SA-β-Gal staining, and the decrease in CD4 T cells were observed in skin with overexpressed WTAP.

## Discussion

Here, we presented a pivotal role of WTAP-mediated N6-methyladenosine RNA modification in skin aging, particularly through its regulation of ELF3 and subsequent upregulation of IRF8. Our findings revealed a novel mechanistic pathway in cellular senescence, highlighting the critical function of WTAP in skin tissue homeostasis and aging. The observed upregulation of WTAP in aging skin tissues and its impact on HDFs underscores its potential as a biomarker and therapeutic target in combating skin aging.

Skin aging is a complex process in which HDF senescence plays a crucial role 39-41. Recent studies reveal that m6A modification regulates multiple skin disorders, including skin inflammation and photoaging 42-45. m6A methylation is dynamically regulated by methyltransferase and demethylase, which have opposite catalytic effects, respectively 12, 21, 26. Previous studies have verified that the dysregulation of FTO and METTL3 contributed to cell senescence by upregulating m6A levels of GCs cells 46 and fibroblast-like synovial cells (FLS) 47. Our study revealed WTAP is a key inducer of skin aging. WTAP, involved in forming the m6A methylation complex, plays a role in various biological processes by regulating target gene expression in an m6A-dependent manner. In this study, we identified ELF3 as a key target gene of WTAP. ELF3 (E74 Like ETS Transcription Factor 3), a member of ETS transcription factor family, involved in the biological processes such as differentiation, proliferation, and migration in mesenchymal stem cells, immune cells, and vascular endothelial cells 48-50. Dysregulated transcription factors have been shown to influence cell senescence via inhibiting longevity gene transcription regulation or activating senescence-related risk factors, these will lead to the aging of organisms 41. Although the relationship between ELF3 and senescence remains unclear, other ETS transcription factors have been reported to be extensively involved in the process of cellular senescence. In a cholangiocyte senescence model, the ETS1 transcription factor was found to induce cell senescence by upregulating cyclin-dependent kinase inhibitor 2A (CDKN2A/p16INK4a) expression 51. Moreover, ERK/ETS1 signaling axis involved in the aging process of hematopoietic stem cells (HSC) 52. Here, we found that upregulated ELF3 in senescent HDFs involved WTAP-induced cell senescence.

Several previous studies have shown that chronic inflammation can affect the progression and pathological development of cellular aging and body aging through the senescence-associated secretory phenotype (SASP). Senescent cells secrete several extracellular factors leading to SASP that can reinforce senescence and amplify inflammation 53, 54. Interferon is part of the immune defense against viral infection, but at the same time, activation of the interferon pathway adversely affects various aging tissues and organs in mammals. Interferon regulatory factor 8, a key regulator of interferon gene, promotes cellular senescence and SASP secretion in various cells 38, 55. In this study, we confirmed that IRF8 is a direct target, involving in ELF3-induced cell senescence. However, it is not clear whether ELF3, as a transcription factor, can extensively regulate other downstream molecules to mediate the senescence of HDFs, and further studies are needed to provide a full understanding.

Looking forward, our study sets the stage for further exploration into the multifaceted roles of WTAP in skin aging biology. Skin, as the largest organ, is a primary site of interaction with the environment and is significantly influenced by immunological changes 56. As skin ages, its ability to interact effectively with immune cells, including T cells, is altered. This alteration in interaction can be partly attributed to the changes in the skin's microenvironment, impacting the functionality and distribution of immune cells, including CD4 T cells 57. One critical area of future research involves understanding m6A-modification in various skin cell types beyond HDFs, particularly immune cells. As skin aging is intricately linked with immune responses, investigating m6A-modification's role in these cells could provide significant insights into the complex interplay between skin aging and immunological changes. Figure S1 shows that WTAP is not only mainly expressed in HDF, but also expressed in immune cells. Therefore, we speculate that local overexpression of WTAP in the skin may also be involved in skin organ aging by affecting immune cells. Additionally, further studies should focus on the potential of WTAP-mediated m6A modification as a target for anti-aging therapies. Such research could lead to novel interventions for skin aging and age-related skin disorders.

In summary, this study demonstrates the role and mechanism of WTAP in cellular senescence. WTAP promotes the expression of ELF3 in m6A modification, thereby inducing IRF8-mediated SASP in senescence cells. Our study reveals the pro-senescence activities of WTAP in HDFs, providing a rationale for exploring WTAP-mediated m6A modification as a target for anti-aging treatments in skin.

## Supplementary Material

Supplementary figures and tables.

## Abbreviations

WTAP: Wilms' tumor 1-associating protein
ELF3: E74 like ETS transcription factor 3
IRF8: Interferon regulatory factors 8
HDF: Human dermal fibroblasts
SASP: Senescence-associated secretory phenotype
m6A: N6-methyladenosine
MeRIP-qPCR: m6A RNA-IP-qRT-PCR
ChIP: Chromatin Immunoprecipitation
