# Supplementary material for: WTAP Mediated N6-methyladenosine RNA Modification of ELF3 Drives Cellular Senescence by Upregulating IRF8
Source: Int J Biol Sci. 2024 Feb 25;20(5):1763–77. doi: 10.7150/ijbs.90459 (PMC10929181; doi:10.7150/ijbs.90459)
Supplement: Supplementary file 1 — Supplementary figures and tables. [file ijbsv20p1763s1.pdf]

Supporting information for

WTAP Mediated N6-methyladenosine RNA Modification of ELF3 Drives Cellular Senescence by Upregulating IRF8

Lei Zhou[2,3 †], Yun Zhong[1,2 †], Fan Wang[1,2], Yi Guo[1,2], Rui Mao[1,2], Hongfu Xie[1,2], Yiya Zhang[1,2,4 *], Ji Li[1,2,4 *]

Address:
1. Department of Dermatology, Xiangya Hospital, Central South University, Changsha, P.R. China
2. Hunan key laboratory of aging biology, Xiangya Hospital, Central South University, Changsha, P.R. China
3. Department of Dermatology, the Third Affiliated Hospital, Sun Yat-sen University, Guangzhou, P.R. China
4. National Clinical Research Center for Geriatric Disorders，Xiangya Hospital, Central South University, Changsha, Hunan, P.R. China, 410008

† These authors contributed equally to this work.
* Corresponding author.
Ji Li: liji_xy@csu.edu.cn, Tel: +86 731 84327472;
Yiya Zhang: yiya0108@csu.edu.cn

Keywords: WTAP; m6A; senescence; ELF3; IRF8

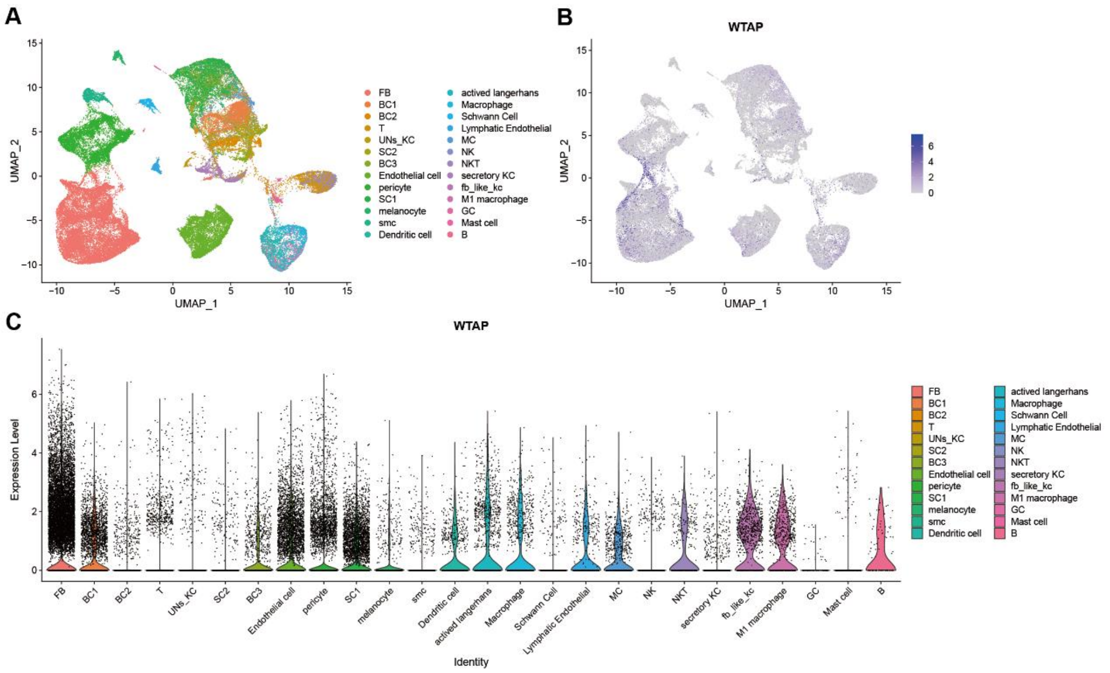

Figure S1

WTAP Expression Profile Across Skin Cell Types by scRNA-Seq Analysis. A. Diplot of single cell clustering; B. Featureplot of WTAP; C. Vlnplot of WTAP in each cell. FB, Fibroblasts; BC, Basal Cells; B, B Cells; T, T Cells; KC, Keratinocytes; UNs_KC, Undefined Keratinocytes; SMC, Smooth Muscle Cells; MC, Mast Cells; NK, Natural Killer Cells; NKT, Natural Killer T Cells; GC, Germinal Center cells.

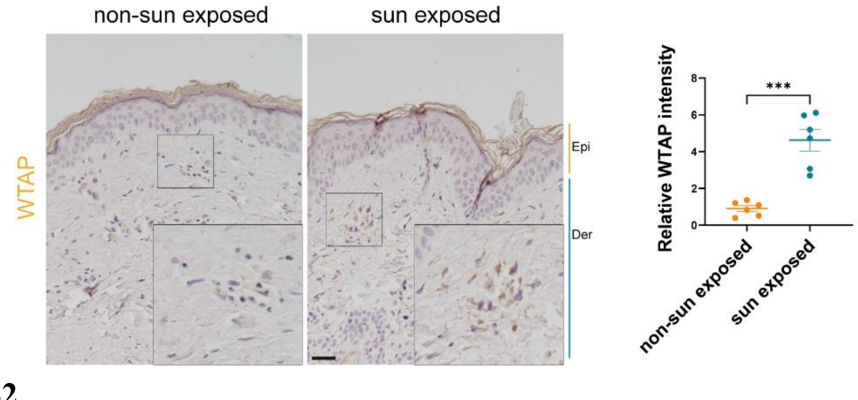

Figure S2

Immunohistochemistry staining of WTAP in human skin of non-exposed (n=8), and exposed areas (n=6). Data are shown as mean ± SEM. Epi, epidermis; Der, dermis. Scale bars, 50μm. ***P < 0.001.

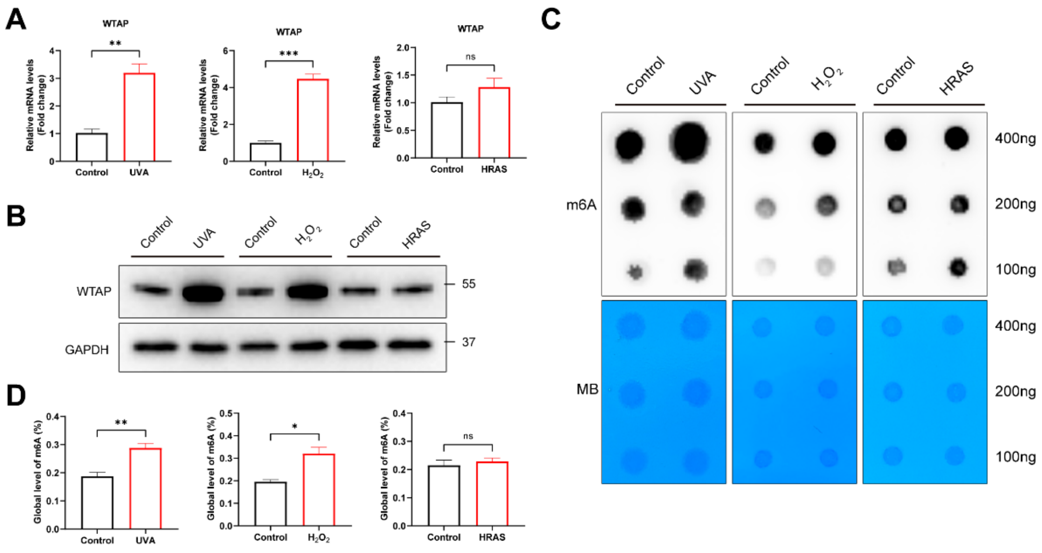

Figure S3

WTAP expression and m6A modification was upregulated in premature senescent HDFs. (A) qPCR and (B) western blotting showed the expression of WTAP in hydrogen peroxide (H_2O_2), UVA, or HRAS oncogene induced HDFs. Overall m6A modification in hydrogen peroxide (H_2O_2), UVA, or HRAS oncogene induced HDFs by Dot Blot (C) and m6A colorimetric assay (D); Methylene blue staining was used as loading control. Data are shown as mean ± SEM. $^*P < 0.05$; $^{**}P < 0.01$; $^{***}P < 0.001$; ns, not significant.

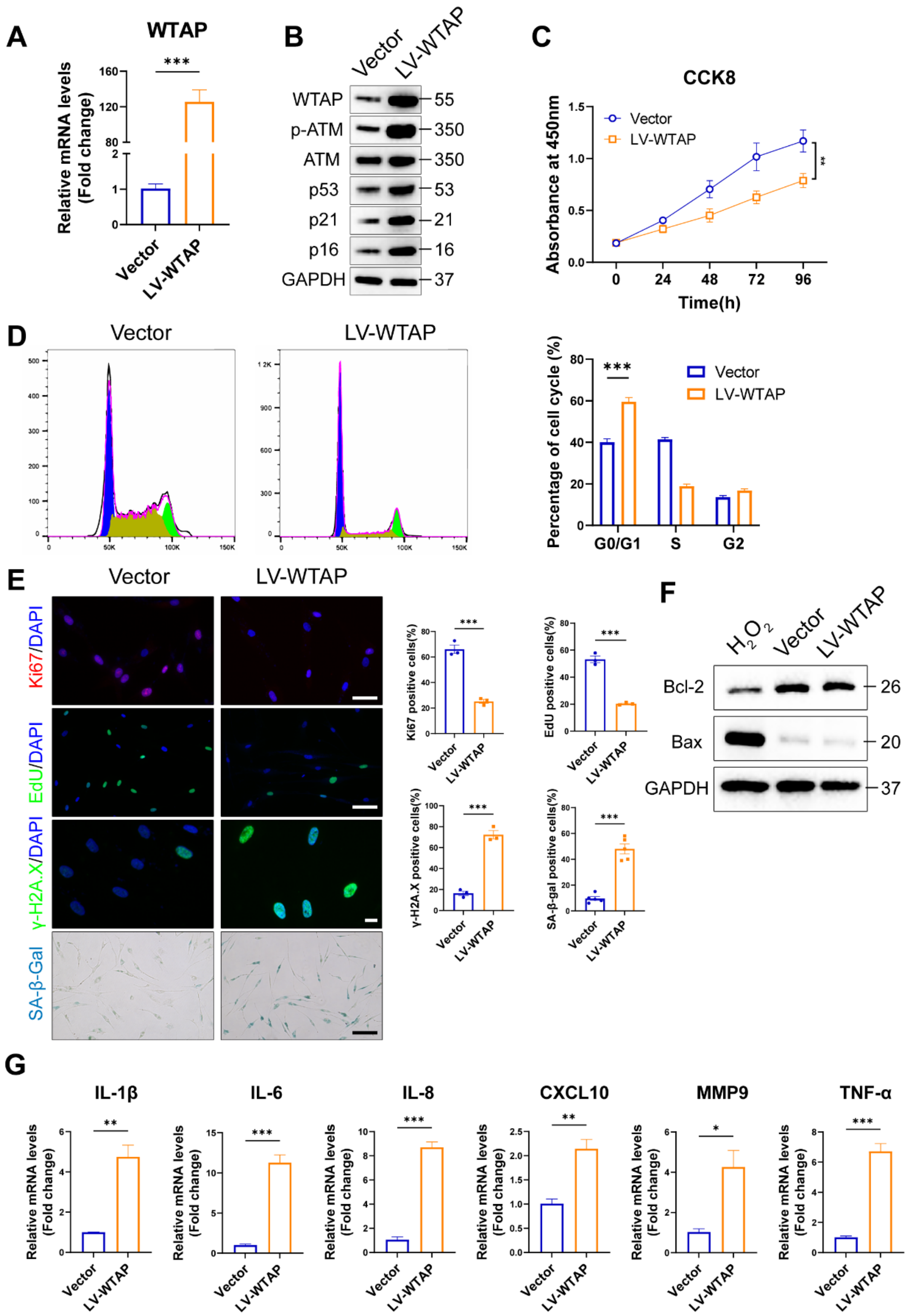

Figure S4

Ectopic expression of WTAP induced cellular senescence in young-passages HDFs. Young-passages HDFs (PD < 10) were infected with WTAP or negative control lentivirus. (A), The LV-WTAP upregulated the mRNA expression of WTAP. (B), The

proteins levels of WTAP, p-ATM, ATM, p53, p21, and p16 by western blotting. (C), Cell proliferation measured by CCK8. (D), Cell cycle analysis measured by flow cytometry. (E), Immunofluorescence staining of Ki67, Edu, and γ-H2A.X, SA-β-Gal staining in HDFs upon shRNA-mediated knockdown of WTAP. Scale bar, 50 μm. (F), The proteins levels of bcl-2 and bax by western blotting. (G), The mRNA expression levels of IL-1β, IL-6, IL-8, CXCL10, MMP3 and TNF-α by RT-qPCR. Data are shown as mean ± SEM. *P < 0.05; **P < 0.01; ***P < 0.001.

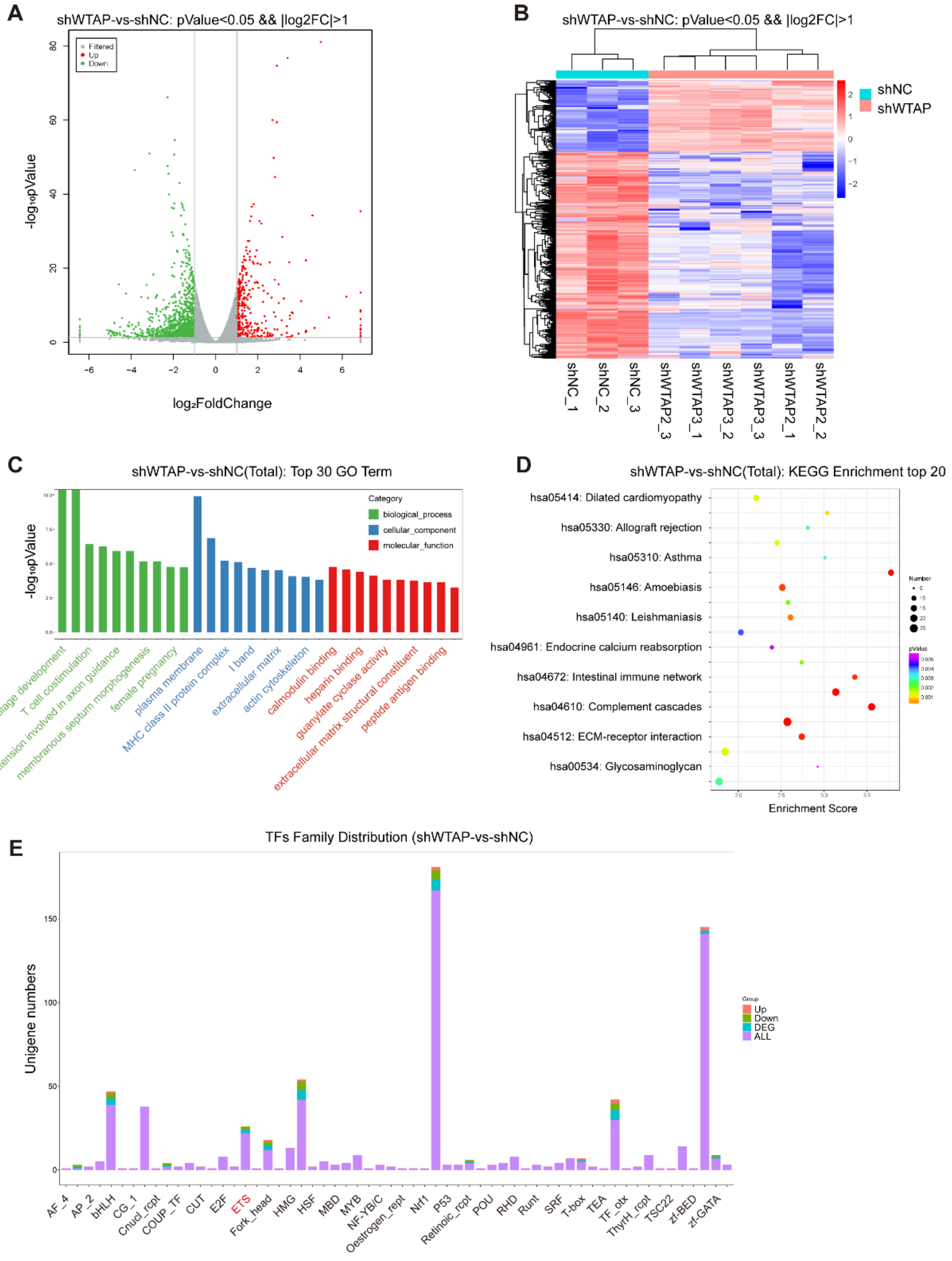

Figure S5

RNA-seq results revealed the DEGs (Differentially expressed genes) between HDFs-shWTAP and HDFs-shNC. (A, B) The volcano map and heatmap of DEGs. High or low expression is indicated by red or green in the digram. (C), GO analysis indicated the biological processes (BP), cellular components (CC), and molecular functions (MF) of DEGs. (D), KEGG pathway of DEGs. (E), Differential transcription factor family distribution.

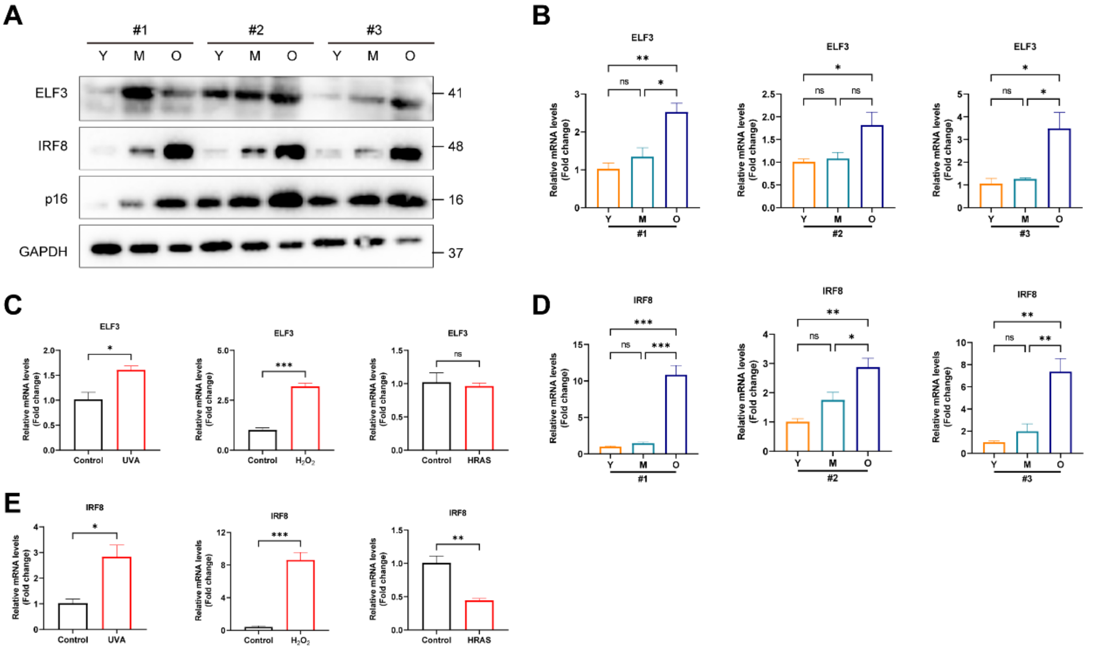

Figure S6

ELF3 and IRF8 expression were upregulated in the different cellular senescence models. (A) western blot assay and (B, D) qPCR assay revealed the ELF3 and IRF8 expression in young, middle-aged, and old HDFs of different individuals. (C, E) qPCR assay revealed the ELF3 and IRF8 expression in hydrogen peroxide (H_2O_2), UVA, or HRAS oncogene induced HDFs. Data are shown as mean ± SEM. *P < 0.05; **P < 0.01; ***P < 0.001; ns, not significant.

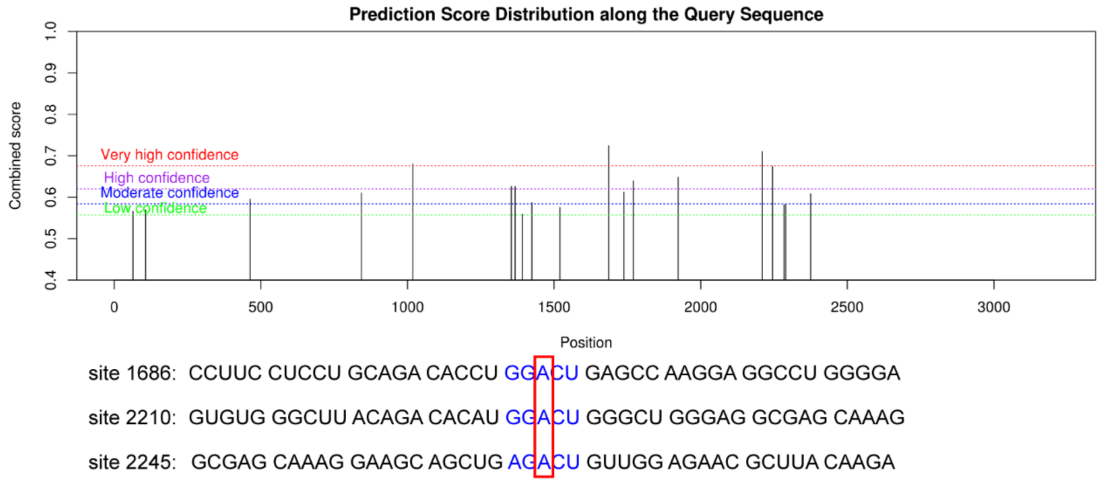

Figure S7

The high confidence m6A sites of ELF3 were predicted in a motif-dependent m6A site predictor SRAMP (http://www.cuilab.cn/sramp).

Appendix Table 1. Details of each dataset.

	Subject ID	Sex	Age at Biopsy
GSE138669	SC1nor	Male	63
	SC4nor	Male	54
	SC18nor	Female	66
	SC32nor	Female	23
	SC33nor	Female	62
	SC34nor	Male	24
	SC50nor	Male	64
	SC68nor	Female	48
	SC124nor	Male	54
	SC125nor	Male	61
GSE156326	skin1	Female	30
	skin2	Female	36
	skin3	Female	43
GSE162183	Ctrl1	Female	32
	Ctrl2	Male	23
	Ctrl3	Male	47
GSE130973	y1	na	25
	y2	na	27
	o1	na	53
	o2	na	70
	o3	na	69
aging_2018	SC1control	male	63
	SC14control	male	54
	SC18control	female	66
	SC32control	female	23
	SC33control	female	62

	SC34control	male	24

Appendix Table 2. Information on skin samples from individuals of different ages.

Sample ID	Sample classification	Age/Gender	Lesion area
1	Young	19/F	Upper limbs
2	Young	22/M	Abdomen
3	Young	22/M	Upper limbs
4	Young	23/M	Upper limbs
5	Young	24/F	Back
6	Young	26/M	Back
7	Young	26/M	Back
8	Young	28/F	Back
9	Middle-aged	31/M	Back
10	Middle-aged	32/M	Upper limbs
11	Middle-aged	32/F	Back
12	Middle-aged	33/M	Upper limbs
13	Middle-aged	34/F	Upper limbs
14	Middle-aged	39/M	Back
15	Middle-aged	41/M	Abdomen
16	Middle-aged	42/F	Upper limbs
17	Middle-aged	48/M	Back
18	Middle-aged	50/M	Abdomen
19	Middle-aged	59/F	Upper limbs
20	Middle-aged	63/F	Chest
21	Old	71/M	Back
22	Old	75/M	Back
23	Old	76/M	Abdomen
24	Old	78/F	Chest
25	Old	78/F	Upper limbs
26	Old	81/M	Back

Appendix Table 3. List of primer sequences in this study.

Gene symbol	Forward primer	Reverse primer
**Primers for RT-qPCR:**		
Human-GAPDH	GGAGCGAGATCCCTCCAAAAT	GGCTGTTGTCATACTTCTCATGG
Human-WTAP	CTTCCCAAGAAGGTTCGATTGA	TCAGACTCTCTTAGGCCAGTTAC
Human-ELF3	GGCCGATGACTTGGTACTGAC	GCTTGCGTCGTACTTGTTCTTC
Human-IRF8	ATGTGTGACCGGAATGGTGG	AGTCCTGGATACATGCTACTGTC
Human-IL-1β	ATGATGGCTTATTACAGTGGCAA	GTCGGAGATTCGTAGCTGGA
Human-IL-6	ACTCACCTCTTCAGAACGAATTG	CCATCTTTGGAAGGTTCAGGTTG
Human-IL-8	TTTTGCCAAGGAGTGCTAAAGA	AACCCTCTGCACCCAGTTTTC
Human-CXCL10	GTGGCATTCAAGGAGTACCTC	TGATGGCCTTCGATTCTGGATT
Human-MMP3	AGTCTTCCAATCCTACTGTTGCT	TCCCCGTCACCTCCAATCC
Human-TNF-α	CCTCTCTCTAATCAGCCCTCTG	GAGGACCTGGGAGTAGATGAG
Mouse-Gapdh	AGGTCGGTGTGAACGGATTTG	TGTAGACCATGTAGTTGAGGTCA
Mouse-Il1β	GCAACTGTTCCTGAACTCAACT	ATCTTTTGGGGTCCGTCAACT
Mouse-Il1β	GCAACTGTTCCTGAACTCAACT	ATCTTTTGGGGTCCGTCAACT
Mouse-Il6	CTGCAAGAGACTTCCATCCAG	AGTGGTATAGACAGGTCTGTTGG
Mouse-Tnf-α	CAGGCGGTGCCTATGTCTC	CGATCACCCCGAAGTTCAGTAG

Mouse-Mmp9	CTGGACAGCCAGACACTAAAG	CTCGCGGCAAGTCTTCAGAG
Mouse-Tgfb	CTCCCGTGGCTTCTAGTGC	GCCTTAGTTTGGACAGGATCTG
Mouse-p16	CGCAGGTTCTTGGTCACTGT	TGTTCACGAAAGCCAGAGCG
**Primer for shRNA:**		
shNC	CCGGCAACAAGATGAAGAGCACCAACTCGAGTTGGTGCTCTTCATCTTGTTGTTTTTG	AATTCAAAAACAACAAGATGAAGAGCACCAACTCGAGTTGGTGCTCTTCATCTTGTTG
shWTAP#2	CCGGGCAAGAGTGTACTACTCAAATCTCGAGATTTGAGTAGTACACTCTTGCTTTTTG	AATTCAAAAAGCAAGAGTGTACTACTCAAATCTCGAGATTTGAGTAGTACACTCTTGC
shWTAP#3	CCGGGGCAAGTACACAGATCTTAACCTCGAGGTTAAGATCTGTGTACTTGCCTTTTTG	AATTCAAAAAGGCAAGTACACAGATCTTAACCTCGAGGTTAAGATCTGTGTACTTGCC
shELF3#1	CCGGCCGAAAGCTGAGCAAAGAGTACTCGAGTACTCTTTGCTCAGCTTTCGGTTTTTG	AATTCAAAAACCGAAAGCTGAGCAAAGAGTACTCGAGTACTCTTTGCTCAGCTTTCGG
shELF3#2	CCGGGCCATGAGGTACTACTACAAACTCGAGTTTGTAGTAGTACCTCATGGCTTTTTG	AATTCAAAAAGCCATGAGGTACTACTACAAACTCGAGTTTGTAGTAGTACCTCATGGC
shIRF8#1	CCGGCAGTAGCATGTATCCAGGACTGATTCTCGAGAATCAGTCCTGGATACATGCTACTGTTTTTG	AATTCAAAAACAGTAGCATGTATCCAGGACTGATTCTCGAGAATCAGTCCTGGATACATGCTACTG
shIRF8#2	CCGGGGAAACACGCTGGCAAGCAAGATTACTCGAGTAATCTTGCTTGCCAGCGTGTTTCCTTTTTG	AATTCAAAAAGGAAACACGCTGGCAAGCAAGATTACTCGAGTAATCTTGCTTGCCAGCGTGTTTCC
**Primer for plasmids or promoter:**		
LV-WTAP	CCGGAATTCCGGATGACCAACGAAGAACCT	GCTCTAGAGCTTACAAAACTGAACCCTGTACA
LV-ELF3	CCGGAATTCCGGATGGCTGC	TGCTCTAGAGCATCAGTTCC

	AACCTGTGAG	GACTCTGGAG
LV-IRF8	CCGGAATTCCGGATGTGTGACCGGAATGGT	GCTCTAGAGCTTAGACGGTGATCTGTTGGT
IRF8-promoter	CGGGGTACCCCGTGCCTCCCGATATCTGAGG	CCGCTCGAGCGGACTGTGCCTACCTGCCGCCGT
**Primers for IRF8 promoter site-directed mutagenesis:**		
ELF3 binding site1 mutation:	GTTTAAGCCAGacgaTGTAGTCTTG	CAAGACTACAtcgtCTGGCTTAAAC
ELF3 binding site2 mutation:	CACCTGCCCCAgcgaATTTTAGACCA	TGGTCTAAAATtcgcTGGGGCAGGTG
**Primers used for ChIP in the IRF8 promoter:**		
Distant region:	TGTAATTCCAGAGAATAAAGAA	CGCGGGACACGGGCGCCCT
ELF3 binding site1:	CACTATATCTAGCACATAGTAG	TTCTGCTCTTATTTAATGGGTA
ELF3 binding site 2:	ATAACGCGCGGCGGGCATCACT	CCCGCCGGTCTCCGCCTTCACG
